# Potential Interactions Between Soil-Transmitted Helminths and Herpes Simplex Virus Type II: Implications for Sexual and Reproductive Health in Sub-Saharan African

**DOI:** 10.3390/biology13121050

**Published:** 2024-12-15

**Authors:** Roxanne Pillay, Pragalathan Naidoo, Zamathombeni Duma, Khethiwe N. Bhengu, Miranda N. Mpaka-Mbatha, Nomzamo Nembe-Mafa, Zilungile L. Mkhize-Kwitshana

**Affiliations:** 1 Department of Biomedical Sciences, Faculty of Natural Sciences, Mangosuthu University of Technology, Umlazi, Durban 4031, South Africa; 2 Department of Medical Microbiology, College of Health Sciences, School of Laboratory Medicine & Medical Sciences, Nelson R. Mandela School of Medicine, University of KwaZulu-Natal, Durban 4001, South Africa; 3 Division of Research Capacity Development, South African Medical Research Council (SAMRC), Tygerberg, Cape Town 7505, South Africa; 4 Department of Biomedical Sciences, University of Johannesburg, Doorfontein Campus, Johannesburg 2028, South Africa; 5 Biomedical Sciences Department of Life and Consumer Sciences, College of Agriculture and Environmental Sciences, University of South Africa, Florida Campus, Johannesburg 1710, South Africa

**Keywords:** soil-transmitted helminths, herpes simplex virus-2, co-infection, immunological interactions, sub-Saharan Africa

## Abstract

Sub-Saharan Africa (SSA) bears a disproportionate and overlapping burden of soil-transmitted helminths (STHs) and sexually transmitted viral infections. An estimated 232 million pre-school and school-aged children in SSA are vulnerable to STH infections. Together with this, SSA has a high prevalence of herpes simplex virus type II (HSV-2), the primary cause of genital herpes. Studies have examined the immunological interactions between STHs and human immunodeficiency virus and human papillomavirus during co-infections. However, epidemiological and immunological studies on STH-HSV-2 co-infections are lacking, therefore their impact on sexual and reproductive health is not fully understood. STH-driven Th2 immune responses are known to downregulate Th1/Th17 immune responses. Therefore, during STH-HSV-2 co-infections, STH-driven immune responses may alter host immunity to HSV-2 and HSV-2 pathology. Herein, we provide an overview of the burden of STH and HSV-2 infections in SSA, and host immune responses to STH and HSV-2 infections. Further, we emphasize the relevance and urgent need for (i) focused research into the interactions between these important pathogens, and (ii) integrated approaches to improve the clinical detection and management of STH-HSV-2 co-infections in SSA.

## Simple Summary

In sub-Saharan Africa (SSA), soil-transmitted helminths (STHs) and herpes simplex virus type II (HSV-2) infections are widespread, and co-infections are likely to occur. It is against this backdrop, that we examine the potential interactions between STHs and HSV-2. STHs and HSV-2 induce opposing host immune responses—HSV-2 triggers a proinflammatory T-helper 1 (Th1) immune response, whereas STHs induce dominant anti-inflammatory T-helper type 2 (Th2) immune responses. It is well known that STHs have bystander effects on unrelated conditions by downregulating Th1 and T-helper type 17 (Th17) immune responses. In this way, STHs may potentially compromise essential anti-HSV-2 Th1 responses, leading to enhanced susceptibility to HSV-2 and HSV-2 pathology. Surprisingly, there is a significant lack of epidemiological and immunological studies on STH-HSV-2 co-infections in humans. Thus, we highlight the need for studies that focus on STH-HSV-2 co-infections in SSA.

## 1. Introduction

Sub-Saharan Africa (SSA) has the second largest population size of approximately 1.3 billion people, comprising 15.5% of the total global population [1]. The continent bears a disproportionate burden of sexually transmitted viral infections [human immunodeficiency virus (HIV), human papillomavirus (HPV), and herpes simplex virus type II (HSV-2) [2] as well as soil-transmitted helminth (STH) infections [3]. The most prevalent STHs are *Ascaris lumbricoides*, *Trichuris trichiura*, *Necator americanus*, and *Ancylostoma duodenale*. These STHs typically cause chronic and asymptomatic infections and are associated with significant morbidity [4]. The co-endemicity of STHs and sexually transmitted viral infections can have significant consequences for sexual and reproductive health. While there is evidence of how STHs influence HIV [5], and HPV [6,7,8], the effect of STHs on HSV-2 is poorly understood. Insight into the effect of STHs on HSV-2 co-infections is relevant, particularly in SSA, where both pathogens are highly prevalent. Herein, we provide an overview of the burden of STH and HSV-2 infections. Drawing from existing literature on the local and/or systemic effects of STHs on viral co-infections, we postulate the potential effects of STHs on HSV-2 during co-infection. Finally, we offer recommendations for future studies and therapeutic interventions aimed at addressing STH-HSV-2 co-infections and their impact on sexual and reproductive health in SSA.

## 2. Literature Search

A literature search was conducted on PubMed and Google Scholar to identify studies related to STH and HSV-2 mono- and co-infections and their impact on host immunity. The following search terms were used: “soil-transmitted helminths”, “soil-transmitted helminths and immune responses”, “HSV-2”, “HSV-2 and immune responses”, and “soil- transmitted helminths and HSV-2 co-infection”. Experimental studies, human studies, and review articles in English were retrieved. No year or region restrictions were applied.

## 3. STH Infection Burden

Helminths are macroparasitic worms that have infected humans for thousands of years. Evidence of their clinical manifestations has been documented in Hippocrates, Egyptian, and Bible records, and helminth eggs and protozoan cysts have been identified in coprolites and other naturally and/or artificially preserved fossils [9,10]. Helminths are grouped into two major categories: nematodes or roundworms and platyhelminths or flatworms. Nematodes comprise STHs, also known as intestinal helminths, and filarial parasites. While STHs cause intestinal infections, filarial parasites cause lymphatic filariasis (elephantiasis) and onchocerciasis (river blindness) [9]. Flatworms comprise trematodes (flukes) and cestodes (tapeworms) [10,11].

STHs are widely distributed parasitic worms, causing more than 1.5 billion human infections globally. Most infections occur in East Asia, China, SSA, and the Americas [3]. The three main medically important STHs are roundworms (*Ascaris lumbricoides*), whipworms (*Trichuris trichiura*), and hookworms (*Necator americanus* and *Ancylostoma duodenale*) [3,12]. Table 1 summarizes the classification, biology, and global prevalence of the major human STHs discussed in this review.

Key drivers of STH infection include poverty, overcrowded living spaces, insufficient clean water resources, poor hygiene practices, and inadequate sanitation [3]. In endemic regions, humidity and warm soil temperatures favor the development of non-fertile eggs into either fertile eggs (*Ascaris lumbricoides* and *Trichuris trichiura*) or infective larvae (hookworms) [24]. Infection with roundworms (*Ascaris lumbricoides*) and whipworms (*Trichuris trichiura*) occurs when embryonated eggs from contaminated water or food sources are ingested. In hookworm infections (*Necator americanus* and *Ancylostoma duodenale*), infective larvae penetrate the skin. Once inside the host, the parasites undergo several developmental stages, including migration of larvae through host tissues, maturation into adult worms, mating, and release of eggs back into the environment via the feces of the infected host [12].

Typically, STHs cause asymptomatic infections and are associated with higher morbidity than mortality [4]. Chronic infections and heavy worm burdens are associated with malnutrition and anemia, stunted physical growth, and impaired intellectual, cognitive, and educational development [12]. Severe complications associated with heavy worm burdens include rectal prolapse (trichuriasis), intestinal blockage or perforation, and duct blockages that may lead to biliary or pancreatic disease (ascariasis). Each year, children die from intestinal obstructions or STH-associated complications that require surgical intervention, which is not always available in resource-poor regions of SSA [25,26,27].

Although highly prevalent, STHs are classified as neglected tropical diseases (NTDs) due to three distinguishing features: (i) infections primarily occur in resource-poor tropical and subtropical developing countries, (ii) infections are chronic and insidious in nature, and (iii) the impact of infection burden on educational and economic development has not been quantified [12,14]. In SSA, STHs are the most widespread NTDs, contributing to 85% of the NTD burden [28]. Table 2 summarizes the prevalence of STHs in the different countries in SSA.

Those most vulnerable to STHs include preschool-aged children (pre-SAC; 1–4 years), school-aged children (SAC; 5–14 years), girls and women of reproductive age, pregnant and/or breastfeeding women, and people with high-risk occupations, such as miners or tea-pickers living in STH-endemic regions [3].

To mitigate the risk of infection, the World Health Organisation (WHO) introduced widespread deworming and preventive chemotherapy (PC) programs in STH-endemic regions. The regular administration of anthelmintic drugs, primarily mebendazole and albendazole, to at-risk children, reduces both the number of moderate- and heavy-worm burden infections, and the overall prevalence of STH infections [12,31].

There has been a significant improvement in the WHO efforts to prevent and eliminate STHs in SSA. For example, one of the WHO 2020 NTD Roadmap targets was to treat a minimum of 75% of at-risk SAC (5–14 years) in helminth-endemic regions where infection prevalence exceeded 20%. In SSA, approximately 70% of at-risk children received regular anthelmintic treatment by 2018, and there was a notable reduction in the prevalence of STH infection in SAC, from 44% in 2000 to 13% in 2018 [32]. Despite this progress, several barriers may hinder the successful elimination of STHs. For example, recurring infections with *Ascaris lumbricoides* and *Trichuris trichiura* have been reported [33]. In addition, interruptions, such as those caused by the recent COVID-19 pandemic, hinder the successful implementation of PC programs. To focus on control measures for COVID-19, some countries in SSA, including South Africa, Malawi, Botswana, Namibia, Lesotho, Eswatini, and Gabon, did not implement their PC for SAC in 2021 [31]. Importantly, sustainable and effective PC programs require an integrated approach that combines clean water supplies, improved sanitation, health education, behavior change, and the regular implementation of PC [12,31,32,33].

## 4. HSV-2 Infection Burden

The Herpesviridae family comprises three subgroups, namely alpha-, beta-, and gamma-herpesviruses. Herpes simplex virus (HSV) is a double-stranded, human alpha-herpesvirus, with two known serotypes: HSV type I (HSV-1) and HSV type II (HSV-2). HSV-1 infection primarily causes corneal keratitis and/or oral blisters, whereas HSV-2 infection is associated with genital herpes and genital ulcer disease [34].

HSV-2 is among the most prevalent sexually transmitted viral infections worldwide. In 2016, the total number of HSV-2 infections among people aged 15–49 years worldwide, was approximately 491.5 million (13.2% global prevalence). In 2016, the annual incidence of HSV-2 among people aged 15–49 years worldwide, was approximately 23.9 million [35]. HSV-2 prevalence varies significantly across the WHO regions. Infection rates are notably highest in areas with low socio-economic status and limited healthcare resources, particularly within SSA. The estimated HSV-2 seroprevalence in SSA is 33%, substantially exceeding rates in other regions, such as 7% in Europe and 17% in the Americas (Table 3). There is also considerable variation in HSV-2 prevalence among SSA subregions, with the highest rates observed in Eastern and Southern Africa, followed by Central and Western Africa. Moreover, infection is more prevalent among women than men, both in Africa and globally [35,36].

HSV-2 infects the genital mucosa and replicates in keratinocytes that line the epithelium, leading to genital lesions [37]. Furthermore, by establishing latency in sensory neurons and ganglia, HSV-2 can evade the immune system and antivirals, thereby causing lifelong infections [38]. Although HSV-2 primarily causes self-limiting and asymptomatic infections, both symptomatic and asymptomatic individuals actively shed the virus during reactivation periods. Lifelong reactivation of genital lesions commonly occurs in symptomatic individuals [39]. Rarely, HSV-2 is associated with life-threatening conditions, such as herpes simplex encephalitis in newborns and immunocompromised individuals [39].

Despite its high prevalence, there are currently no prophylactic or curative treatments for HSV-2. While antiviral treatment with drugs, such as acyclovir, reduces recurrent HSV-2 infections, they are ineffective in eliminating viral shedding. Furthermore, resistance to antivirals, especially in immunocompromised individuals, has been reported [40].

HSV-2 is recognized as a critical driver of the HIV epidemic in various regions, most notably in SSA, where the highest HSV-2 and HIV infection burdens are recorded [41]. For example, Bradley et al. [42] reported that among individuals infected with HSV-2, the HIV prevalence was 41%, whereas among those without HSV-2 infection, the HIV prevalence was 6% [42]. This striking epidemiological association between HSV-2 and HIV infection has significant public health consequences. HSV-2 infection is also known to increase the risk of transmitting and acquiring HIV infection by almost three-fold. Biologically, HSV-2 infection facilitates HIV transmission by causing genital ulcers, which compromise the integrity of the genital mucosal barrier. In addition, HSV-2 infections cause increased trafficking of Th1 CD4^+^ cells to the infected genital site, thereby increasing the number of available target cells for HIV entry [43,44]. Individuals with incident HSV-2 infection are more vulnerable to HIV infection [45,46]. Furthermore, the interactions between HSV-2 and HIV are bidirectional. HSV-2 co-infection promotes HIV genital shedding [47] and may accelerate the progression of HIV disease [48,49], whereas HIV co-infection associates with increased occurrence and quantity of HSV-2 viral shedding [50].

HSV-2 has also been linked to cervical cancer; however, its role in cervical carcinogenesis is poorly understood. To date, a few studies have examined the association between HSV-2 and cervical cancer, with conflicting results [51]. There is considerable disparity in the global distribution of cervical cancer [52,53], and resource-poor regions across the world (South-East Asia, Melanesia, SSA, and South America) carry approximately 90% of the cervical cancer disease burden. SSA has the highest incidence of cervical cancer cases and the highest cervical cancer mortality rates. In addition, most cancer-related deaths among women in SSA are due to cervical cancer [53].

Longstanding infection with oncogenic or high-risk HPV strains (mainly HPV-16 and HPV-18) is the leading risk factor for cervical cancer [54,55]. However, in 90% of women, high-risk HPV infection does not progress to cervical cancer [54]. This implies that co-factors, such as environmental, host, and viral factors, may act jointly with HPV to contribute to cervical carcinogenesis. Other well-known risk factors for cervical cancer include smoking, multiple sexual partners, long-term oral contraceptive use, and HIV infection [52,54]. Because of its persistent nature and ability to reactivate from latency, HSV-2 may cause abnormal cellular DNA replication and genetic mutations. Therefore, persistent infection with HSV-2 could lead to abnormal differentiation and proliferation of the cervical epithelium, which may lead to cervical cancer [56]. Furthermore, HSV-2 promotes ulceration and inflammation of the genital epithelium and induces an influx of Th1 CD4^+^ cells to the infected site. In addition to promoting the acquisition of HIV, HSV-2-induced genital ulceration may facilitate infection with oncogenic strains of Epstein–Barr virus (EBV) and HPV [57].

There is epidemiological evidence supporting the association between HSV-2 and cervical cancer [58,59,60]. Li and Wen [60] reported that HSV-2 infection was linked to the occurrence of cervical cancer, even after controlling for oncogenic HPV as a confounding factor. In addition, their study found that co-infection with HPV and HSV-2 correlated with a higher relative risk for cervical cancer compared to a single infection with either virus [60]. Similarly, Zhao et al. [58] observed that while HSV-1 was not associated with cervical cancer, HSV-2 infection—either alone or in combination with HPV—was linked to cervical carcinogenesis [58]. Koanga et al. [59] further demonstrated that HSV infection promoted cervical inflammation and abnormalities in infected women, suggesting it may serve as a co-factor in cervical carcinogenesis [59]. These findings underscore the potential link between HSV-2 and cervical carcinogenesis, which warrants further investigation. Given its high prevalence and associations with HIV and HPV, HSV-2 represents a significant public health challenge, particularly in SSA.

## 5. Immune Response to STHs

STHs exhibit notable differences in their routes of infection and lifecycles and demonstrate distinct variations in tissue tropism. Hookworms, for example, infect the human host via skin penetration, whereas *Ascaris lumbricoides* and *Trichuris trichiura* are orally infective [15,16]. Moreover, STHs have complex lifecycles, comprising different stages. For example, the larval stages of *Ascaris lumbricoides* complete extra-intestinal migratory phases during which larvae hatch in the intestine, and migrate through the liver and lungs, before finally settling in the small intestine, where they develop into adult worms and reproduce. As they transit through the different tissues, they induce local inflammation, cellular remodeling, and immune modulation. Similarly, the hookworms (*Ancylostoma duodenale* and *Necator americanus*) undergo extra-intestinal migration via blood circulation and the lungs, before establishing themselves in the small intestine. In contrast, *Trichuris trichiura* does not exhibit tissue migration, establishing infection in the large intestine [15,16]. STH developmental stages and tissue migrations occur over time, spanning weeks to years, depending on the STH species and its specific mammalian host. Thus, host immunity is often shaped differently based on the tissue involved or possibly the STH’s lifespan [61]. These differences contribute to varying clinical outcomes as observed across STH infections. In addition, the pathological effects of STHs are linked to the worm burden and whether the infection is acute or chronic [61]. Despite these species-specific differences, STHs have been shown to influence and regulate the host immunity (i.e., parasite-specific immunoregulation), by typically inducing dominant anti-inflammatory Th2 and immunomodulatory responses. STH-driven immune responses reduce STH-associated inflammation and favor the long-term survival of both the host and parasite. A dominant Th2 phenotype consequently downregulates Th1 and Th17 proinflammatory immune responses, limits worm burden, and promotes healing of damaged host tissue [62]. Secretion of alarmin cytokines, [Interleukin (IL)-25, IL-33, and thymic stromal lymphopoietin (TSLP)], initiates a coordinated Th2 response. IL-25 and IL-33 subsequently activate Th2 innate lymphoid cells (ILC2s), which secrete IL-5 and IL-13. TSLP suppresses IL-12, a Th1 response cytokine, and supports dendritic cell (DC) maturation [63]. IL-4, IL-5, and IL-13 amplify the Th2 response within the intestine and stimulate immunoglobulin-E (IgE) and immunoglobulin-G (IgG) antibody production by activated B cells. Furthermore, Th2 cytokines recruit and activate various myeloid cells (basophils, eosinophils, macrophages, and mast cells). Alternatively-activated macrophages (AAMs) and eosinophils attempt to trap and kill the worms as they migrate through host tissues [63]. Worm expulsion is further promoted by the “weep and sweep” mechanism, which includes increased mucus secretion by goblet cells and the concerted action of various Th2 cytokines (IL-3, IL-4, IL-5, IL-13, and IL-9), CD4^+^ T cells, ILC2s, and myeloid cells (basophils, eosinophils, and mast cells) [64].

**Figure 1:**
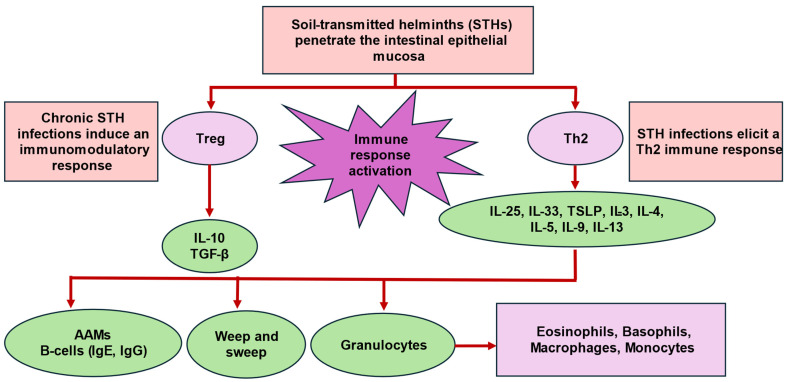
Illustration of the immune response to STHs. Footnote: AAMs: alternatively-activated macrophages; IgE: immunoglobulin E; IgG: immunoglobulin G; IL: interleukin; STH: soil-transmitted helminth; TGF-β: transforming growth factor beta; Th2: T-helper type 2 cells; Treg: regulatory T cells; TSLP: thymic stromal lymphopoietin.

During chronic STH infections, immunomodulation is further enhanced by regulatory T (Treg) cells, which secrete immunosuppressive cytokines, such as IL-10 and transforming growth factor beta (TGF-β) [62] (Figure 1).

Moreover, STHs alter host immunity, directly or indirectly, by employing several strategies, including (i) the induction of host resistance and tolerance responses, (ii) secretion of immunomodulatory products, such as excretory-secretory products (ESP), and (iii) interaction with the intestinal microbiome [65]. Interestingly, the STH-induced immune effects are not limited to the tissues they transit through or colonize, but extend to distal parts of the body, leading to systemic immunomodulation. Importantly, opposing immune responses are directed against STHs (i.e., Th2 and immunomodulatory) versus viral and bacterial pathogens (i.e., Th1 and Th17 immunity), and these responses broadly inhibit one another. Consequently, this may lead to varied outcomes in terms of host health and susceptibility to co-infections. Epidemiological data also indicate an inverse relationship between STHs and the incidence of allergies, autoimmune diseases, and metabolic disorders. Additionally, STHs may alter responses to vaccines [reviewed in [61,66,67]].

Co-infections with multiple helminths are also common, particularly in endemic regions. As observed in animal studies, poly-helminth infections can impact the outcome of each infection in several ways: (i) one helminth infection can suppress the immune response to a concurrent helminth infection; (ii) the timing of each infection impacts the resulting immunopathological effects; and (iii) host susceptibility or resistance may be altered by the co-infecting helminth, independent of immunity developed through immunization [67].

## 6. Immune Response to HSV-2

**Figure 2:**
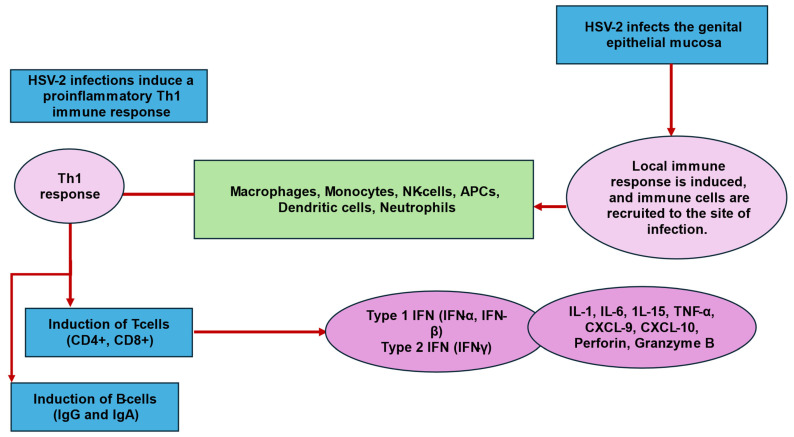
Illustration of the immune response to HSV-2 infection. Footnote: APCs: antigen-presenting cells; CXCL: chemokine (C-X-C motif) ligand; HSV-2: herpes simplex virus type II; IFN: interferon; IFN-α: interferon alpha; IFN-β: interferon beta; IFN-γ: interferon gamma; IL: interleukin; NK: natural killer; Th1: T-helper type 1 cells; TNF-α: tumor necrosis factor alpha.

An effective Th1 proinflammatory immune response against HSV-2 requires multiple coordinated mechanisms of both innate and adaptive components of the immune system (Figure 2).

Firstly, innate immunity is activated and is essential for early control of the virus and for initiating the adaptive arm of immunity [68,69]. Following infection, toll-like receptors (TLRs) present on infected genital epithelial cells detect and bind to HSV-2-specific pathogen-associated molecular patterns (PAMPs) [69]. Several immune cells participate in the innate response to HSV-2 infection. These include antigen-presenting cells (APCs), plasmacytoid dendritic cells (pDC), neutrophils, macrophages, monocytes, and natural killer (NK) cells [39]. Once activated, TLRs stimulate the synthesis of type I interferons (IFN-α and IFN-β), which inhibit translation and promote the degradation of HSV-2 mRNA. TLRs also stimulate the production of proinflammatory cytokines (IL-1, IL-6, and TNF-α). Simultaneously, type I IFNs stimulate the maturation of dendritic cells (DC) and IL-15 synthesis, which supports NK cell production and survival. NK cells secrete IFN-γ, a type II interferon, perforin and granzyme B, which subsequently stimulate apoptosis of HSV-2-infected cells. IFN-γ further enhances the anti-HSV-2 response through the activation of the inducible nitric oxide synthase gene (iNOS gene) [69].

Secondly, both cell-mediated and humoral components of adaptive immunity are essential for viral clearance. Cell-mediated immunity is initiated by an influx and activation of Th1 CD4^+^ T cells at the infected genital site. Th1 CD4^+^ cells are activated when they bind to MHC class II receptors present on local APCs, such as pDCs. Following activation, CD4^+^ T cells secrete IFN-γ, which stimulates genital epithelial cells to release the chemokines, CXCL9 and CXCL10, thereby creating a chemokine gradient. Subsequently, cytotoxic CD8+ T cells are attracted to the infected genital mucosa, and cause epithelial cells and APCs to release nitric oxide (NO) [69]. CD8^+^ T cells also secrete IFN-γ, which further promotes apoptosis of HSV-2-infected cells via perforin- and fas-mediated cytolytic mechanisms [70].

During the humoral-mediated immune response, activated B-cells release antibodies (IgG and IgA). However, how antibody-mediated responses contribute to HSV-2 immunity is debatable because viral glycoproteins released by HSV-2 evade antibody-mediated protection. Lastly, although Treg cells occur at the infected site, their precise role in HSV-2 clearance is yet to be clearly defined [68,69].

## 7. Impact of STHs on HSV-2 and Other Viral Co-Infections

As highlighted earlier, STHs induce systemic immunomodulatory responses that have downstream effects on bacterial, protozoan, and viral co-infections. In the context of STH-viral co-infections, studies have examined the effects of STHs on enteric, respiratory, hepatotropic, and sexually transmitted viruses (reviewed recently by [67,71]). Despite the significant burden and geographic overlap of STHs and HSV-2, there are no epidemiological and immunological studies on human STH-HSV-2 co-infections in SSA. Therefore, the true impact of STH-HSV-2 co-infections remains unclear. In this section, we have focused on STH-viral co-infections. Drawing from recent murine and human studies, where available, we provide key examples of the influence of STHs on viral co-infections, emphasizing the existing research gaps in relation to human STH-HSV-2 co-infections.

Co-infection with STHs may lead to beneficial or detrimental outcomes for viral infections. Moreover, STH and viral interactions may occur in a bidirectional manner, subsequently affecting both pathogens [67]. To date, our understanding of how STHs impact viral co-infections is shaped primarily by studying animal models, which provide valuable mechanistic insight into STH-viral co-infections.

Animal models of STHs, such as *Nippostrongylus brasiliensis*, *Heligmosomoides polygyrus, Ascaris suum, Trichinella spiralis,* and *Trichuris muris*, are widely used because of their close similarities in lifecycle to their human counterparts [72].

In the case of STH-enteric viral co-infections, STHs impair host immune responses to enteric viruses. For example, acute infection with *Heligmosomoides polygyrus* and *Trichinella spiralis* exacerbated enteric murine norovirus (MNV) in co-infected mice [73]. This impaired antiviral response was independent of changes in the intestinal microbiota, dependent on STAT6-induced activation of alternative macrophages (AAM), and was partially restored following neutralization of Ym1, a molecule of AAM [73]. Similar results were observed in mice co-infected with *Heligmosomoides polygyrus* and West Nile virus (WNV). This phenotype was associated with enhanced WNV pathology, increased gut dysmotility and permeability, compromised CD8^+^ T cell responses, and higher mortality. Poorer outcomes in co-infected mice were mediated by STH-induced Th2-specific tuft cell and IL25/IL4 signaling pathways [74]. Co-infection with *Heligmosomoides polygyrus* enhanced the replication and shedding of murine astrovirus [75].

STH co-infection has also been shown to impair host immune responses to some respiratory viruses. As an example, poorer outcomes were observed in mice co-infected with *Ascaris suum* and vaccinia virus; this enhanced pathology was associated with the ablation of CD8^+^ T cells, significantly reduced IFN-γ-secreting CD4^+^ T cells, higher viral loads, and increased mortality. Moreover, co-infected mice had markedly fewer *Ascaris* lung-stage larvae, suggesting a two-way interaction between *Ascaris suum* and the vaccinia virus [76]. In support of these findings, *Ascaris*-infected pigs were more susceptible to respiratory viral infection [77]. In a human cohort of Colombian infants, *Ascaris*-specific IgE levels and TLR-4 gene polymorphisms were associated with more severe respiratory syncytial virus (RSV) bronchiolitis [78].

Interactions between herpesvirus and STHs have also been studied. In one study, acute infection with *Heligmosomoides polygyrus* or *Schistosoma mansoni eggs*, a trematode helminth, mediated the reactivation of human Kaposi’s sarcoma-associated herpesvirus in vitro [79]. Consistent with these findings, in vivo co-infection with *Heligmosomoides polygyrus* or *Schistosoma mansoni eggs* reactivated murine gammaherpesvirus (MHV-68) infection from latency. MHV-68 reactivation required a “two-signal” mechanism involving IL-4-induced STAT6 activation and IFN-γ-blockage [79].

In some instances, STHs are beneficial to the outcomes of viral co-infections, particularly enhancing protection against respiratory viruses. *Nippostrongylus brasiliensis* and *Heligmosomoides polygyrus* enhanced the host immune response to murine gammaherpesvirus 4 (MHV-4) respiratory infection through an IL-15-driven expansion of virtual memory CD8^+^ T cells [80]. In another study, co-infection with *Heligmosomoides polygyrus* conferred antiviral protection against pulmonary RSV; this STH-induced protection was associated with reduced pulmonary inflammation and pathology in co-infected mice, and was driven by microbiota-dependent production of type 1 IFN [81]. *Trichinella spiralis* also enhanced protection against influenza virus by reducing virus-associated lung inflammation in co-infected mice, without altering viral replication or clearance [82]. More recently, studies have focused on the effects of STHs on the novel SARS-CoV-2 virus and its associated disease COVID-19. In some cases, SARS-CoV-2/COVID-19 are associated with exaggerated proinflammatory Th1 responses that may lead to life-threatening severe acute respiratory failure and systemic inflammatory syndrome. It has been hypothesized that STH-induced anti-inflammatory and immunosuppressive Th2 responses may downregulate SARS-CoV-2/COVID-19-associated Th1 responses, thus mitigating severe SARS-CoV-2 and COVID-19 outcomes in STH-endemic regions [83]. Moreover, STH-induced immunomodulation may reduce the expression of ACE-2, a critical receptor for SARS-CoV-2 entry into cells, thereby potentially impairing viral entry and replication [83]. In support of these hypotheses, helminth co-infection (*Hymenolepis nana, Schistosoma mansoni,* and *Trichuris trichiura*) mitigated COVID-19 severity in patients from Africa [84].

The distribution and prevalence of STHs and sexually transmitted viral infections overlap geographically. For example, the high prevalence of STHs in HIV-infected individuals [85,86,87] and HPV-infected women [6] has been reported. In addition, previous studies have demonstrated that STHs regulate host immune mechanisms related to sexually transmitted viral co-infections [6,8,85,88,89,90]. Previous studies have demonstrated a link between STHs and HIV co-infections, including lower CD4^+^ counts [88], dysregulated immune cells, and higher HIV viral loads [85]. In the case of HPV, Gravitt et al. [6] reported that HPV infection was more prevalent in older women, aged 30–45 years, who were co-infected with STHs. Notably, although STHs neither enter nor reside in the vaginal tract, the authors detected IL-4, a Th2 cytokine, in the cervical fluids collected from STH-infected women. In addition, they reported that IL-4 levels were positively associated with other cytokines typically produced during antihelminth immune responses (IL-5, IL-8, IL-10, IL-21, IL-25, and IL-31), suggesting that higher HPV prevalence among STH-infected women may be driven by STH-induced immunomodulation, which may impair effective host anti-HPV control [6]. Similarly, in other human studies, hookworm infections were correlated with a higher risk of HPV infection [7], higher HPV viral loads, and distinct mixed Type 1/Type 2 immune profiles in the vaginal tracts of women with HPV and hookworm co-infections [8]. Conversely, human cervical cells infected with *Nippostrongylus brasiliensis* in vitro, demonstrated decreased uptake of HPV16 pseudovirions, reduced cell migration, and lower expression levels of vimentin and N-cadherin, which are key epithelial-to-mesenchymal transition (EMT) markers used to evaluate cancer progression [91]. In addition, in vivo infection of mice with *Nippostrongylus brasiliensis* was associated with reduced vimentin expression in the vaginal tract, thereby further demonstrating the parasite’s potential to impair cervical cancer progression [91]. When taken together, these co-infection studies demonstrate both favorable and unfavorable effects of STHs on HPV and/or cervical cancer [6,91], however, they are insufficient to conclusively define the role of STHs in HPV co-infection and cervical cancer. Therefore, further studies on the influence of STHs on female genital tract-specific immunity to HPV and the development of cervical cancer are needed.

In summary, the above studies highlight important features of STH-viral co-infections: (i) interactions may occur in a bidirectional manner with potential consequences for both STHs and viruses; and (ii) STHs may have favorable or unfavorable effects on viral co-infections; whether co-infection outcomes are beneficial or detrimental to the host depends on one or more key factors, including the type of STH, the stage of STH lifecycle, tissue tropism, the type of viral infection, and the timing and site/s of infection [67,71]. Considering these factors, it is likely that interactions between STHs and HSV-2 are complex because various factors may contribute to their interactions.

**Figure 3:**
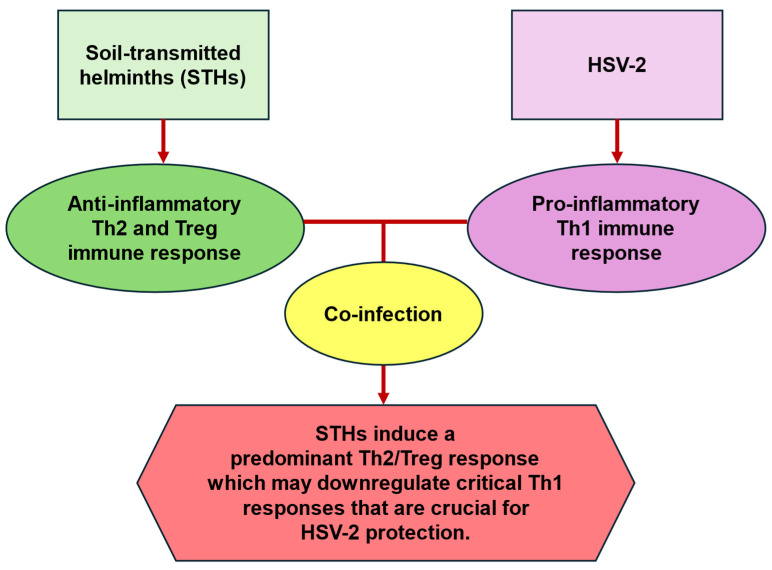
Illustration of potential immune response during STH-HSV-2 co-infection. Footnote: HSV-2: herpes simplex virus type II; STHs: soil-transmitted helminths; Th1: T-helper type 1 cells; Th2: T-helper type 2 cells; Treg: regulatory T cells.

However, there is a scarcity of epidemiological and immunological studies on the impact of STH-induced Th2 immune responses on host immunity to HSV-2 infection, and currently only data from a single animal study exists [89]. In their murine model, Chetty et al. [89] demonstrated that acute intestinal infection with *Nippostrongylus brasiliensis*, which closely resembles human hookworms, stimulated a Th2 profile in the vaginal tract. This acute *Nippostrongylus brasiliensis* infection was associated with enhanced vaginal ulceration and pathology following HSV-2 co-infection. Although this enhanced HSV-2 pathology was independent of IL-4 receptor alpha (IL4ra), it was driven by IL-5 and associated with higher eosinophil counts and IL-33 levels in the vaginal tract [89]. The findings by Chetty et al. [89] suggest that the systemic STH-induced Th2 immune profile, detected in the murine vaginal tract, impaired host immunity to HSV-2, thereby exacerbating HSV-2 pathology. In support, Oh et al. [92] reported that dysbiosis of vaginal microbiota led to increased levels of IL-33 in the murine vagina, which subsequently impaired antiviral responses to HSV-2. Importantly, Chetty et al. [89] demonstrated that although STHs neither enter nor reside in the vaginal tract during their lifecycles, they were able to induce potent Th2 immune responses specific to the vaginal tract. These findings are consistent with previously observed clinical correlations between STHs and sexually transmitted viral infections in vaginal tissue [6,8]. The underlying mechanisms by which *Nippostrongylus brasiliensis* influences the vaginal tract need further investigation. Mechanisms such as the ability of STHs to induce host resistance and tolerance responses, alter the microbiome, as well as the immunomodulatory capabilities of their ESP, may likely be contributory factors. For example, it has been suggested that STH-derived ESP may promote immune cell trafficking and Th2 responses in uncolonized tissues, such as the vaginal tract [93]. We argue that the work by Chetty et al. [89] provides a foundation for future human studies, raising important questions regarding (a) the mechanisms by which STHs elicit such changes in the non-colonized vaginal tract, and (b) the implications for anti-HSV-2 immunity, HSV-2 progression and pathology in STH-HSV-2 co-infected individuals. Given that STHs stimulate Th2-biased immune responses, whereas protection from HSV-2 requires a Th1 response, we hypothesize that STHs may compromise host immunity to HSV-2 during co-infection (Figure 3). This could enhance susceptibility to HSV-2, promote viral persistence and pathology, and impair responses to HSV-2 treatment. Likewise, STH-HSV-2 co-infections could have significant consequences for sexual and reproductive health, such as increased risk of acquiring other sexually transmitted infections, infertility, and cancer progression [94]. In such scenarios, poorly resourced healthcare systems in SSA, in terms of diagnosis, treatment, and long-term management, would be negatively impacted. In addition, no studies have examined the effects of HSV-2 co-infection on STH lifecycle, such as larval migration, adult worm establishment, persistence in the intestine, and anti-STH immunity. These are important research gaps that warrant further investigation to understand the association between STHs and HSV-2 and their impact on overall health.

## 8. STHs Eradication and HSV-2 Management Experiences in Africa

The WHO-recommended HSV-2 treatment regime includes (i) primary HSV-2 infection, (ii) recurrent HSV-2 infection, and (iii) frequent and/or severe recurrent HSV-2 infection. The mainstay HSV-2 antivirals include acyclovir and valacyclovir [95], which inhibit nucleoside analog-polymerase and pyrophosphate analog-polymerase and act against all types of herpesviruses [96]. Without an effective vaccine for HSV-2, these recommended antivirals act by reducing the number of recurrent HSV-2 infections but cannot eliminate HSV-2 shedding. Furthermore, drug resistance to antivirals due to chronic use and among immunocompromised individuals has been reported [40].

Treatment recommendations by the WHO for STH-endemic regions include the use of broad-spectrum anthelmintics. Mebendazole (500 mg) and albendazole (400 mg) are administered orally as single doses to treat STHs [3]. These benzimidazole agents bind to parasite β-tubulin and inhibit parasitic microtubule polymerization, with subsequent death of worms within several days [12]. In regions where STHs and schistosomiasis are co-endemic, the WHO recommends that praziquantel (for schistosomiasis) and mebendazole/albendazole are safely administered together. This is currently being done, particularly in SSA, where most of these infections occur [97].

Anthelmintics are affordable, effective in treating infections, safe, and easy to administer at regular intervals [12]. However, STH PC efforts are hindered by frequent re-infections that occur because of the high numbers of STH eggs and larvae in the environment [33]. In addition, there are growing concerns about drug-resistant helminths, which may further reduce the efficacy of anthelmintics [98,99,100]. As an alternative to anthelmintics, anti-STH vaccines are considered effective strategies to combat STHs in the long term. Despite several efforts in anti-STH vaccine development over the last decade, there are currently no approved human anti-STH vaccines [reviewed in [101,102]]. Moreover, the development of effective anti-STH vaccines is thwarted by several challenges. These include the complex biology and lifecycles of STHs, multiple stages, and stage-specific antigen expression within the same host, and the immunomodulatory properties of STHs [101,102].

Due to the significant burden of HIV/AIDS in SSA, research into the effect of anthelmintic treatment on host immunity to sexually transmitted viral infections has primarily focused on HIV. For example, deworming of STH-HIV co-infected individuals was shown to decrease HIV viral loads and improve CD4^+^ cell counts [87,103,104]. However, data on STH co-infection with other viruses, particularly HSV-2, are lacking. For STH-HSV-2 co-infections, there are currently no general guidelines for therapeutic interventions other than treating each infection following diagnosis [67]. To date, the effect of anthelmintic treatment on HSV-2 co-infection has not been investigated. Likewise, the effect of HSV-2 treatment on STH co-infection has yet to be investigated. However, the interactions between STHs and HSV-2 may need to be considered for both anti-HSV-2 treatment and deworming programs. As previously mentioned, STH co-infections can alter the immune response in a manner that supports the onset and progression of concurrent HSV-2 infections [89]. Thus, it follows that treatment of concurrent HSV-2 might be more effective if the STH infection has been treated prior to, or in parallel, if there are no concerns about adverse drug interactions. Therefore, long-term studies are needed to determine the safety and efficacy of anthelmintic treatment to control STHs in HSV-2 co-infected individuals. Moreover, as there is currently no evidence, targeted studies investigating the impact of therapeutic interventions in STH-HSV-2 co-infections are needed to develop consensus guidelines for the treatment of co-infections. In SSA, integrated treatment and management approaches that actively address STH-HSV-2 co-infections may help to effectively mitigate the burden of STHs and HSV-2 and improve overall sexual and reproductive health.

## 9. Research Implications and Future Directions

HSV-2 prevalence is higher in SSA than in other regions of the world [35]. However, despite its significant disease burden, there are no effective vaccines to curb HSV-2 transmission. We argue that there is a need for ongoing efforts to identify effective preventive and curative strategies for HSV-2 infection.

The extent and impact of STH-HSV-2 co-infections in SSA is understudied. There are several outstanding research questions regarding whether STHs have beneficial, harmful, or no effects on anti-HSV-2 immunity and HSV-2 pathology. Moreover, whether HSV-2 co-infection has an impact on the STH lifecycle, such as larval migration, adult worm establishment, persistence in the intestine, and anti-STH immunity, has yet to be determined. Although host immune responses to single STH and HSV-2 infections have been extensively examined, human studies that demonstrate whether STH-induced immunity modulates HSV-2-specific immune responses are lacking. It is plausible that the opposing Th1 and Th2 immune responses elicited in HSV-2 and STH infections, respectively, may influence the outcome of HSV-2 infection. Therefore, there is a significant need for studies that examine the burden of STHs and HSV-2 co-infections in SSA. In addition, we assert that multifaceted studies should be undertaken to explore the (a) effects of acute/chronic STH infection on HSV-2 co-infection, (b) identification of STH/HSV-2-specific biomarkers that may serve as diagnostic and/or prognostic markers of infection, (c) impact of STH co-infections on HSV-2 pathology in the reproductive tract, and the implications thereof for susceptibility to HSV-2, and (d) treatment outcomes in STH-HSV-2 co-infections. Focused research on STH-HSV-2 co-infections could provide valuable insights into the mechanistic and immunological interactions between these pathogens. A better understanding of these interactions could inform policymaking and practices in co-endemic regions. For example, screening for HSV-2 in STH-endemic regions might improve patient outcomes by managing co-infections that could exacerbate HSV-2. In addition, the integration of deworming programs with HSV-2 interventions in co-endemic regions could reduce overall disease burden and transmission. Moreover, the investigation of the immune pathways affected by STH-HSV-2 co-infections could help identify new therapeutic targets and guide the development of prevention and control interventions that specifically address co-infected populations. With this view, we assert that integrated approaches that include (1) early diagnosis of STH and HSV-2 single and co-infections, (2) mass deworming programs, (3) ongoing surveillance, (4) health education programs for vulnerable communities, (5) provision of proper sanitation and clean water, (6) development of sensitive diagnostic tools, (7) allocation of resources for multifaceted research, (8) identification of new therapeutic agents, (9) the development of prophylactic vaccines for STH and HSV-2, and (10) the development of consensus guidelines for the treatment of co-infections, are required.

## 10. Conclusions

Host immunity to HSV-2 requires a proinflammatory Th1 immune response, which may be dampened by STHs, which typically elicit opposing Th2 immune responses. Furthermore, chronic STH infections induce immunomodulatory responses that can further reduce immune responses to HSV-2, thereby potentially promoting susceptibility to and enhanced pathology of HSV-2. Given that STHs are widespread in SSA, where HSV-2 is also highly prevalent, the immunomodulatory effects of STHs may influence host immunity to HSV-2. Insight into the interactions between these two important pathogens may improve diagnostic and treatment approaches, guide the design of potential vaccines, and inform policymaking and practices for the clinical detection and management of STHs and HSV-2 in SSA.

## Figures and Tables

**Table 1:** Classification, biology, and global prevalence of the major human STHs in SSA.

STH	Global Prevalence	Classification and Biology	References
*Ascaris lumbricoides*	772–892 million	- Roundworm. - Disease in humans: Ascariasis. - Classification: (i) Domain: Eukaryota, (ii) Kingdom: Animalia, (iii) Subkingdom: Metazoa, (iv) Phylum: Nematoda, (v) Class: Secernentea, (vi) Order: Ascaridida, (vii) Family: Ascarididae, (viii) Genus: *Ascaris*, (viii) Species: *Ascaris lumbricoides*. - Morphology: (i) Unsegmented, cylindrical and elongated bodies. (ii) Worms are white to pinkish in colour. (iii) Adult male: 15–31 cm by 2–4 mm. (iv) Adult female: 20–35 cm by 3–6 mm and produce 240,000 eggs/day. Lifecycle: Embryonated eggs from contaminated soil, food, or water are ingested, moulting into L1, then L2 larvae that penetrate the small intestine. Larvae then migrate through the liver and lungs, where they mature to L3 larvae, ascend the trachea, are swallowed, and re-enter the intestine. There, they moult into L4 larvae and mature into adult worms that reproduce, releasing thousands of eggs per day. Embryonated eggs and adults are viable for several years.	[13,14,15,16,17,18]
*Trichuris trichiura*	429–508 million	- Whipworm. - Disease in humans: Trichuriasis. - Classification: (i) Domain: Eukaryota, (ii) Kingdom: Animalia, (iii) Subkingdom: Metazoa, (iv) Phylum: Nematoda, (v) Class: Adenophorea, (vi) Order: Trichocephalida, (vii) Family: Trichuridae, (viii) Genus: *Trichurus*, (ix) Species: *Trichuris trichiura*. - Morphology: (i) Whip-like appearance. The anterior end is thin and thread-like, while the posterior end is thicker and contains most of the reproductive organs. Males have a coiled tail, whereas females have a straight posterior end. (ii) Worms are white to pinkish in colour. (iii) Adult male: 30–45 mm by 0.1–0.5 mm. (iv) Adult female: 35–55 mm by 0.1–0.5 mm and produce 3000–10,000 eggs/day Lifecycle: Unembryonated eggs from contaminated feces develop into infective eggs in the soil. Infection begins when contaminated soil, food, or water is ingested. The eggs hatch in the small intestine, and L1 larvae migrate to the large intestine, where they penetrate epithelial cells at the crypt base. After several moults, they mature into whip-like adult worms, which can live for years. Eggs remain viable in soil for months under favorable conditions.	[13,14,15,16,19,20,21]
*Necator americanus * and *Ancyclostoma duodenale*	406–480 million	- Hookworms. - Disease in humans: Necatoriasis and Ancylostomiasis, respectively. - Classification: (i) Domain: Eukaryota, (ii) Kingdom: Animalia, (iii) Subkingdom: Metazoa, (iv) Phylum: Nematoda, (v) Class: Secernentea, (vi) Order: Strongylida, (vii) Family: Uncinariidae or Ancylostomidae, (viii) Genus: *Necator* or *Ancylostoma*, (ix) Species: *Necator americanus* or *Ancylostoma duodenale*. - Morphology: (i) Both species have a simple cylindrical body with a curved, hook-like anterior end. (ii) Worms are pale, translucent, or white. (iii) Adult male: 6–11 mm by 0.4–0.6 mm. (iv) Adult female: 10–13 mm by 0.4–0.6 mm and produce 10,000–20,000 (*Ancylostoma duodenale*) and 5000–10,000 (*Necator americanus*) eggs/day. Lifecycle: Eggs released in the host’s feces hatch into rhabditiform (L1) larvae in soil. These larvae feed on organic matter and moult twice to become infective filariform (L3) larvae, which penetrate human skin. Following skin entry, they migrate through the bloodstream to the lungs, ascend the respiratory tract, and are swallowed. In the small intestine, they mature into adult worms, attach to the intestinal lining, and feed on blood. Female worms produce thousands of eggs daily, completing the cycle. Differences in the lifecycles of hookworms: *Necator americanus* remains confined to the intestines after entry and survives for several years, while *Ancylostoma duodenale* larvae can enter dormancy in tissues and reactivate later. Additionally, *Ancylostoma duodenale* can be transmitted through oral ingestion of larvae, unlike *Necator americanus*, which is exclusively acquired via skin penetration. Adult *Ancylostoma duodenale* worms typically survive for several months.	[13,14,15,16,20,22,23]

**Table 2:** Prevalence of STHs in SSA.

Countries in SSA	Total Population, *n*	STH Prevalence	
		*n*	%
Nigeria	232,679,478	48,681,440	20.9
Democratic Republic of Congo	109,276,265	26,830,345	24.6
Ethiopia	132,059,767	27,233,348	20.6
United Republic of Tanzania	68,560,157	24,470,870	35.7
Uganda	50,015,092	19,696,574	39.4
South Africa	64,007,187	13,672,166	21.4
Angola	37,885,849	4,886,509	12.9
Mozambique	34,631,766	12,223,289	35.3
Cameroon	29,123,744	3,493,226	12.0
Madagascar	31,964,956	7,117,949	22.3
Malawi	21,655,286	7,837,505	36.2
Kenya	56,432,944	5,524,519	9.8
Zimbabwe	16,634,373	553,600	3.3
Zambia	21,314,956	4,577,679	21.5
Rwanda	14,256,567	4,218,568	29.6
Sierra Leone	8,642,022	1,189,047	13.8
Togo	9,515,236	2,544,074	26.7
Guinea	14,754,785	2,676,150	18.1
Cote d’Ivoire	31,934,230	2,543,761	8.0
Burundi	14,047,786	969,250	6.9
Benin	14,462,724	1,959,363	13.5
Senegal	18,501,984	1,339,379	7.2
Central African Republic	5,330,690	1,528,494	28.7
Liberia	5,612,817	830,898	14.8
Namibia	3,030,131	330,850	10.9
Congo	6,332,961	839,886	13.3
South Sudan	11,943,408	779,808	6.5
Chad	20,299,123	552,311	2.7
Gabon	2,538,952	465,348	18.3
Lesotho	2,337,423	387,421	16.6
Comoros	866,628	277,694	32.0
Equatorial Guinea	1,892,516	387,783	20.5
Cabo Verde	524,877	143,054	27.3
Guinea-Bissau	2,201,352	383,629	17.4
Botswana	2,521,139	43,831	1.7
Sao Tome and Principe	235,536	83,988	35.7
Gambia	2,759,988	53,098	1.9
Eswatini	1,242,822	20,097	1.6
Ghana	34,427,414	No preventative chemotherapy required	No preventative chemotherapy required
Niger	27,032,412	No preventative chemotherapy required	No preventative chemotherapy required
Overall prevalence, *n* (%)	1,163,487,343 (14.2) ^a^	231,346,801 (19.9) ^b^	

^a^ % Overall prevalence (total population) = total population in sub-Saharan Africa/world population as of 8 November 2024 (8,187,059,938) × 100. Source: [1,29]. ^b^ % Overall prevalence (Helminths prevalence) = total helminths prevalence in sub-Saharan Africa/total population in sub-Saharan Africa × 100. Source: [29,30].

**Table 3:** HSV-2 prevalence in the WHO regions.

WHO Region	Prevalence, *n* (%) ^a^
Africa	162,200,000 (33.0)
The Americas	85,700,000 (17.4)
Eastern Mediterranean	17,900,000 (3.6)
Europe	33,300,000 (6.8)
South-East Asia	86,900,000 (17.7)
Western Pacific	105,500,000 (21.5)
Total	491,500,000 (100)

Estimated numbers (*n*) and proportions (%) of HSV-2 across the WHO regions [data presented are the estimated number of people (*n*) within the 15–49-year age group that were infected with HSV-2 in 2016] ^a^; % proportion = estimated number of HSV-2/estimated number of total HSV-2 × 100. ^a^ Source: Adapted from [35].

## Data Availability

The original contributions presented in this review article are included within the article.
